# Modulation of Frontal Oscillatory Power during Blink Suppression in Children: Effects of Premonitory Urge and Reward

**DOI:** 10.1093/texcom/tgaa046

**Published:** 2020-08-06

**Authors:** Makoto Miyakoshi, Joseph Jurgiel, Andrea Dillon, Susanna Chang, John Piacentini, Scott Makeig, Sandra K Loo

**Affiliations:** 1 Swartz Center for Computational Neuroscience, Institute for Neural Computation, University of California San Diego, La Jolla, CA 92093-0559, USA; 2 Semel Institute for Neuroscience and Human Behavior, University of California Los Angeles, Los Angeles, CA 90095, USA

**Keywords:** blink suppression, children, EEG, reward, urge

## Abstract

There is a dearth of studies examining the underlying mechanisms of blink suppression and the effects of urge and reward, particularly those measuring subsecond electroencephalogram (EEG) brain dynamics. To address these issues, we designed an EEG study to ask 3 questions: 1) How does urge develop? 2) What are EEG-correlates of blink suppression? 3) How does reward change brain dynamics related to urge suppression? This study examined healthy children (*N* = 26, age 8–12 years) during blink suppression under 3 conditions: blink freely (i.e., no suppression), blink suppressed, and blink suppressed for reward. During suppression conditions, children used a joystick to indicate their subjective urge to blink. Results showed that 1) half of the trials were associated with clearly defined urge time course of ~7 s, which was accompanied by EEG delta (1–4 Hz) power reduction localized at anterior cingulate cortex (ACC); 2) the EEG correlates of blink suppression were found in left prefrontal theta (4–8 Hz) power elevation; and 3) reward improved blink suppression performance while reducing the EEG delta power observed in ACC. We concluded that the empirically supported urge time course and underlying EEG modulations provide a subsecond chronospatial model of the brain dynamics during urge- and reward-mediated blink suppression.

## Introduction

Clarifying the neural mechanisms of blink suppression in children is important for understanding how mental effort controls behavior, which may still be under developmental influences, unlike a comparable adult model. This understanding has critical value in child psychiatry, for example, in designing a clinical behavioral training program for treating children with Tourette’s syndrome (Woods and Himle 2004; Greene et al. 2015). These studies reported clinically important findings that although tics had been considered as a result of biological disorder, operant contingencies using a reinforcer ($2 in Woods and Himle 2004) could suppress tic behavior in children. However, the underlying neural mechanism in children remains unclear. Neuroimaging studies during urge suppression help to localize neural substrata and elucidate their dynamics corresponding to the mental processes. One of the earliest studies investigated “air hunger” (or shortness of breath) and found activations in the mid to anterior right insula, a part of the limbic system (Banzett et al. 2000). The most well-studied experimental paradigm to date is blink suppression. Neuroimaging studies using PET on blink suppression reported activation in right insular cortex and anterior cingulate cortex (Lerner et al. 2009). Similarly, functional MRI activations in right insular cortex, right ventrolateral prefrontal cortex, and bilateral temporal gyri showed correlations with a hypothetical model for the time-course of urge. In the model, urge takes 60 s to build up to the peak, after which a blink occurs and is followed by another 15 s to release (Berman et al. 2012). The same group also studied the effect of neurofeedback training using a blink suppression task and reported changes in functional connectivity between anterior insula and medial frontal cortex (Berman et al. 2013). This study was one of several to support the now established relationship between blink suppression and the activation within the right insula.

In addition to insula, other interacting regions, which are mostly distributed in the frontal lobe, have also been implicated in urge suppression. For example, the right ventrolateral prefrontal cortex is another well-established region in response inhibition such as in the Go/No Go task and Stop Signal task (Aron et al. 2004, 2014). The right ventrolateral prefrontal and insular cortices are a part of circuit that maintains volitional suppression of behavior during an increasing sense of urge. A recent study on healthy adults reported the neural correlates of blink suppression to be in bilateral insula, sensorimotor, anterior prefrontal, and parietal cortices, as well as subcortical regions including putamen and caudate (van der Salm et al. 2018). Another study investigated cough suppression after inhaling capsaicin solution (Mazzone et al. 2011). Regions activated included bilateral insula, cingulate cortex, middle frontal gyrus, and posterior cingulate gyrus, which confirmed the involvement of insula in different types of suppression. Developmentally, adults showed more activation in widespread regions during blink suppression compared with children, but blink-suppression-related inhibition in posterior cingulate cortex was relatively comparable (Mazzone et al. 2010). Importantly, they reported bilateral dorsolateral prefrontal cortices (DLPFCs) and anterior cingulate cortex (ACC) to be key regions for both children and adults.

While there is converging evidence from neuroimaging studies, there are still unanswered questions. One critical question remaining is the temporal relationship between increased urge and its associated brain dynamics. As reviewed above, one fMRI study attempted to study the temporal aspect of urge building (Berman et al. 2012). The major limitation in their study was that the hypothetical temporal model of building urge was heuristically determined without empirical data support. Moreover, BOLD signal does not have good temporal resolution compared with electrophysiological measures. In order to answer the question of the relation between urge and brain dynamics, modalities with high temporal resolution such as EEG or MEG are natural choices. However, as far as we know, there have been no EEG studies on the temporal relation between urge and brain dynamics. The other critical question remaining is how reward-facilitated blink suppression is represented in the typically developing brain. It is reported that reward enhances successful tic suppression (Woods and Himle 2004; Greene et al. 2015). However, the neural mechanisms underlying this process are poorly understood. Clarification of this question is particularly important for enhancing behavioral interventions, which are often used for patients with Tourette disorder.

In the present study, we conducted an EEG study of blink suppression performed by healthy control children. The following 3 questions were tested: 1) What is the time course of urge development? 2) What are the EEG-correlates of blink suppression? 3) How does reward change brain dynamics related to urge suppression? To investigate the temporal relation between building urge and brain dynamics, the children used a joystick as an “urgeometer” to indicate their subjective experience of urge. Also, to investigate the effect of reward on urge suppression, there were 3 experimental conditions: 1) Blink Freely/No Suppression (No Supp); 2) Verbal Suppression (Supp); and 3) Suppression for Reward (Supp Rwd). The trials in the latter 2 conditions were subsequently separated into 2 subgroups based on urge (Urge High and Urge Low), and the interaction between urge and reward was tested.

## Materials and Methods

### Sample

Participants were 35 healthy control children between the ages of 8 and 12 years old who were recruited as a comparison group for a larger study on Tourette disorder; data on the patient group will be reported separately. In order to ensure enough trials for the event-related EEG analysis, a minimum threshold of more than 20 blinks in NoSupp condition and 10 blinks in Supp and Supp Rwd conditions (conditions will be described later) was used. The final sample for EEG analysis consisted of 26 children (12 males and 14 females) with a mean age of 9.6 years (SD 1.5, range 8–12). The children were recruited from the community through radio and newspaper advertisements, community organizations, local schools, primary care physicians, and local clinics. After receiving verbal and written explanations of study requirements, and prior to any study procedures, all parents/participants provided written permission and informed consent/assent as approved by the Institutional Review Board.

### Procedures

Subjects were excluded from participation if they were positive for any of the following: presence of any major Diagnostic and Statistical Manual (American Psychiatric Association 2013) Axis I diagnosis or taking any type of psychoactive medication, head injury resulting in concussion, or estimated Full Scale IQ < 80. The absence of psychiatric diagnoses was confirmed using a semistructured diagnostic interview, the Anxiety Disorder Interview Schedule, Child Version (ADIS) (Silverman et al. 2001), which was administered by trained and carefully supervised graduate level psychologists. Estimated intelligence (IQ) was assessed using the Wechsler Abbreviated Scale of Intelligence (WASI) (Weschler 1999).

### Task

There were 3 block-separated conditions: blink freely/no blink suppression (No Supp), verbal instruction for blink suppression (Supp), and blink suppression for reward (Supp Rwd). All children were instructed to blink freely during the No Supp block, while trying to suppress blinks during the 2 blink suppression blocks. During Supp Rwd, children were told that the computer would be counting how many blinks they were able to suppress, and that they would subsequently receive a reward for successful suppression. All children received $10 regardless of how many blinks they exhibited. During the 2 blink suppression blocks, children used a custom joystick to indicate their subjective experience of urge for blinking by moving the stick forward when they felt the urge to blink. The joystick would revert back to the neutral condition automatically once pressure was released. The order of the 3 conditions was counterbalanced across subjects. There were other types of cognitive tasks in between the blink freely/suppression blocks, which will be presented elsewhere. Each block length was between 5 and 7 min.

### E‌EG Recording

EEG signals were recorded using the Electrical Geodesics Incorporated (EGI) hardware and software with 128 Hydrogel electrodes that were embedded in a hydrogel net in an International 10/10 location system. Data were sampled at 1000 Hz and initially referenced to Cz. Electrode-skin impedance threshold was set at 50 kΩ per manufacturer standard for the high input impedance amplifier. Eye movements were monitored by electrodes placed on the outer canthus of each eye for horizontal movements (REOG and LEOG) and by electrodes above the eyes for vertical eye movements. Facial electromyography (EMG) leads were placed on the cheeks bilaterally over the zygomaticus major muscles to assist with detection of facial movements. Key head landmarks (nasion, inion, and preauricular notches) and 3D electrode locations were recorded (Polhemus, Inc.) to allow reconstruction of electrode positions on the scalp. All EEG data were recorded using the Lab Streaming Layer (https://github.com/sccn/labstreaminglayer), which allows integration of multiple data streams including EEG, high-definition video, joy-stick urgeometer, and experimental events.

### E‌EG Preprocessing

Throughout the preprocessing, EEGLAB 14.1.2 (Delorme and Makeig 2004) running under Matlab 2017b (The MathWorks, Inc.) was used. Custom code was written as necessary. There were 2 central signal processing techniques: artifact subspace reconstruction (ASR) (Mullen et al. 2015; Chang et al. 2018, 2019; Gabard-Durnam et al. 2018; Blum et al. 2019; Plechawska-Wojcik et al. 2019), which is an offline version of data cleaning suits from BCILAB (Kothe and Makeig 2013) (see Supplementary Material 7 for detail) and independent component analysis (ICA) (Bell and Sejnowski 1995; Makeig et al. 1996, 1997, 2002). These 2 approaches are complementary in that ASR uses sliding-window principal component analysis (PCA)-based subspace rejection and reconstruction so that it can address data nonstationarity such as infrequent short-lasting bursts by touching electrodes, for example, while ICA can find stationary processes and temporally maximally independent sources such as brain EEG sources as well as nonbrain artifact sources like blink, eye movement, and facial and neck muscle activation by using more sophisticated, physiologically valid assumptions than PCA (Onton and Makeig 2006; Delorme et al. 2012). After preprocessing the scalp recordings with these 2 algorithms, we analyzed event-related spectral perturbation (ERSP) on each anatomically defined source cluster to investigate time–frequency–space decomposed EEG power dynamics related to blink suppression. For full details, see Supplementary Material 1.

### Identifying Blinks

We developed an EEGLAB plugin *countBlinks()* for this project (available from https://sccn.ucsd.edu/eeglab/plugin_uploader/plugin_list_all.php) to manually annotate all the blinks during the tasks by visually examining the time-series data of the independent component (IC) representing blink/vertical eye movement. The principle in this blink identification is peak detection in the EOG-IC time series; hence, the annotated markers refer to the highest-amplitude moment, rather than the onset, of a blink. The solution does not use an algorithm; an annotator judged whether the currently highlighted blink-induced-like EOG waveforms (typically 0.5–1.0 s long) should be labeled as blink or not for each candidate waveform. When the data showed stereotypical blink-induced waveforms, annotating 2–3 blinks per second was possible due to the efficient GUI design. Several automated algorithms were tested out before developing our own solution, but their performances turned out to be often unsatisfactory particularly during blocks with suppression conditions. This was probably because participant’s physical effort to suppress the blinks prevented generation of stereotypical blink-induced EOG waveforms. Thus, we were motivated to instead use manual annotation in an efficient way.

### Statistical Testing

**Figure 1:**
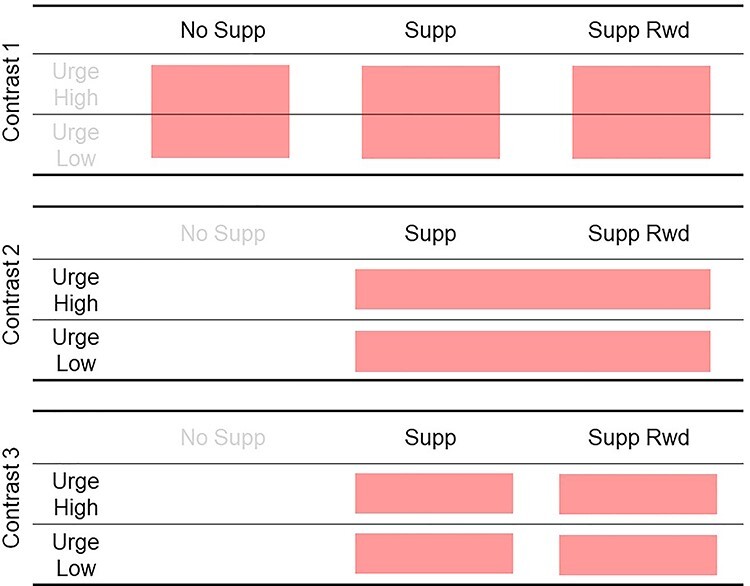
The factorial design of the current study. There were 3 contrasts for which statistical tests were performed. Note that the Contrast 2 and 3 include only 2 suppression conditions because urge data were not collected during “No Supp” condition.

The full factorial design of the current study was 3 suppression factors (No Supp, Supp, Supp Rwd) x 2 urge factors (Urge High, Urge Low), all within-subject design. However, because urge was measured only for the suppression conditions, the No Supp condition did not have urge data. We determined 3 contrasts of interest; Contrast 1, main effect of Suppression; Contrast 2, main effect of Urge; Contrast 3 main effect of Reward (Fig. 1).

Repeated measures ANOVAs were performed for Contrasts 1 and 3, and paired *t*-test for Contrast 2, on each time–frequency pixel of the calculated ERSP tensor with the dimensions of 100 (frequencies, 1 to 55 Hz) × 252 (latency to blink ERP peak, −4030 to 1000 ms) × number_of_ICs (this varies from IC cluster to cluster) for 12 IC clusters. For multiple comparison correction for the 100 × 252 time–frequency points, weak family-wise error rate (wFWER) control was used (Groppe et al. 2011). *t*- or *F*-statistics values were computed for all time–frequency points and thresholded at *P* < 0.001 and *P* < 0.005 for Contrast 1 and Contrast 2, respectively. The true *mass of cluster*, which is the sum of absolute *t*- or *F*-statistics within a time–frequency point cluster, was computed for each cluster. Next, data labels were shuffled, and the same procedure was applied, and the largest mass of cluster was taken to build distribution of surrogate mass of cluster. Finally, 99.9 and 99.5 percentiles of surrogate mass of cluster distribution were determined to be used as a threshold value for omnibus correction. Those true mass of cluster entries that showed larger values than the threshold values were declared to be statistically significant after wFWER control.

## Results

### Behavioral Data

**Figure 2:**
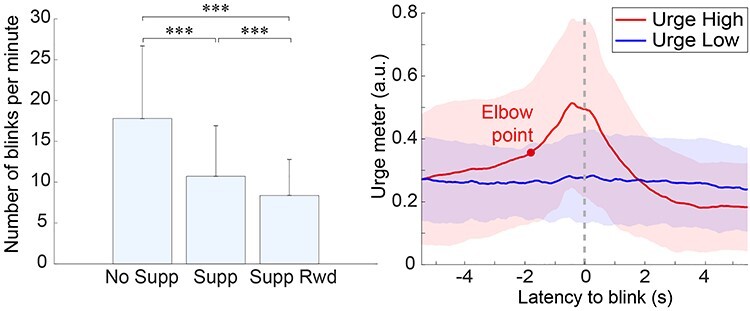
Left, the group-mean number of blink counts for the condition Suppression. The error bar represents 1 SD. ^***^P < 0.001 (Bonferroni-corrected). Right, average time-course of urge input obtained from trials separated into Urge High and Low. The trials were separated into the 2 groups according to the single-trial urge time-series correlation with that of the within-subject mean. The red dot on the Urge High plot around −1.812 s point shows the optimum bisection point that separates the rise of the plot into 2 parts. The color shades in the plot indicate ±1 SD.

The number of blinks was counted for each block and normalized into average counts per minute for each subject. The results were as follows: No Supp, *M* = 17.8 (SD 8.9); Supp, *M* = 10.7 (SD 6.2); Supp Rwd, *M* = 8.4 (SD 4.4). Paired *t*-tests across the 3 conditions confirmed significant reduction of blinks in the order of No Supp, Supp, and Supp Rwd (all *P* < 0.001, Fig. 2). The result confirmed the validity of the experimental control over blink suppression. The distribution of other blinks relative to a blink is shown in Supplementary Material 4.

The grand-mean urgeometer time series (±1 SD) plotted separately for High and Low Urge conditions indicates that urge peak was reached slightly earlier than the EOG-ERP peak latency. The peak latency for Urge High was found at −0.4 s relative to blink EOG-ERP peak. Next, the elbow point of the rising curve up to the peak was obtained using a two-line fitting bisection method to find the point where the residual from the two-line fitting is minimized. Relative to the EOG-ERP peak, the elbow point was found at −1.8 s. The result indicated that the urge increase rate is nonlinear, and it became steeper after −1.8 s. Finally, Urge Low showed a flat pattern, indicating that about half of the blinks (i.e., suppression failures) may have occurred with little to no urge experienced by participants.

For interest, we characterized the impact of eye blinks and urge on sensor-level ERP and their ICA-decomposition. The results are summarized in Supplementary Materials 2 and 3, respectively.

### General Descriptive Statistics about Preprocessing, ICA-Decomposed EEG, and Multiple Comparison Correction

The total amount of variance reduction after all the preprocessing was percent variance accounted for (PVAF) reduction, *M* = 99.7%, *SD* = 0.3, and range 98.6–99.9. This PVAF difference is the result from the following 2 stages of signal processing: reduction to 1.5–55 Hz bandpass filtering and ASR (*M* = 98.4, *SD* = 2.5, and range 88.5–99.9); reduction due to the subsequent IC rejection (*M* = 74.4, *SD* = 13.3, and range 38.5–96.7).

**Figure 3:**
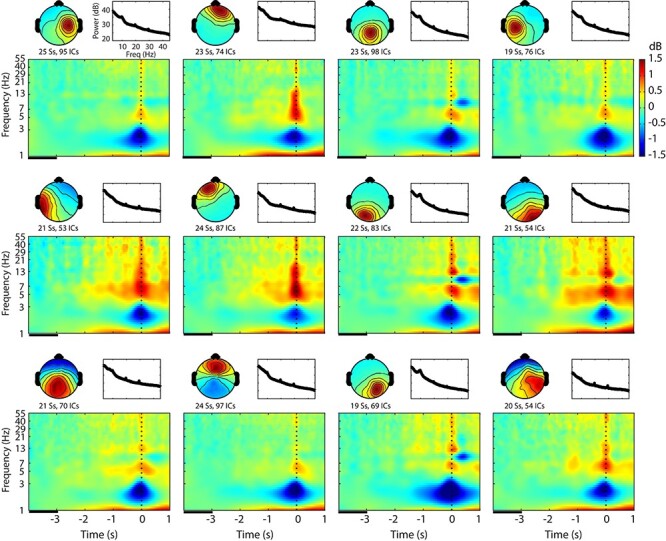
Cluster-mean scalp topography, power spectral density, and event-related spectral perturbation (ERSP) for each of the 12 clusters determined by Silhouette analysis and averaged across all the conditions. This figure shows a general outline of the whole-brain data right after group-level independent component (IC) clustering. The graph scales are identical across the clusters. In the time–frequency plots, baseline period is indicated as a black line between −4 and −3 s relative to blink onset. Ss, subjects; ICs, independent components.

For the group-level analysis, 910/3224 qualified brain ICs were selected from the final sample of 26 participants who showed more than 20 blinks for No Supp and 10 blinks for Supp and Supp Rwd blocks, respectively. The number of brain ICs contributed by individual subjects was *M* = 35.0 (*SD* = 13.8, range 10–61). The optimum numbers of IC clusters based on the spatial coordinates of the dipoles were 12 and 14 for Silhouette and Davies-Bouldin, respectively. Calinski-Harabasz did not show an optimum point. To increase the chance of obtaining a higher number of unique subjects per cluster, we chose to generate 12 IC clusters. Mean scalp topography, power spectral density, and event-related spectral perturbation (ERSP) within the cluster and across all the conditions are shown in Figure 3 to show a general outline of the group-level clustered ICs.

### Main Effect Suppression

**Figure 4:**
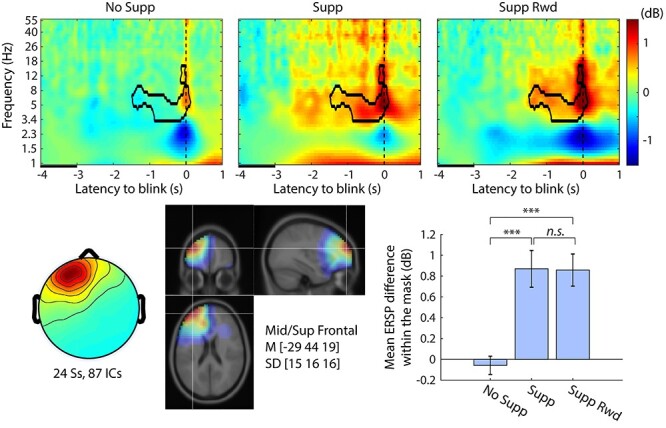
Event-related spectral perturbation (ERSP) plots for main effect Suppression (No Supp, Supp, Supp Rwd) in the left prefrontal independent component (IC) cluster. The contour mask in the time–frequency plots indicates *P* < 0.001 after controlling weak family-wise error rate (wFWER). Top row, ERSP for No Supp, Supp, and Supp Rwd. Baseline period is indicated as a black line between −4 and −3 s relative to blink onset. Bottom left, cluster-mean IC scalp topography. Bottom center, cluster-mean dipole density with FWHM = 20 mm and the centroid coordinate in the MNI template head. Bottom right, the mean ERSP values with SE within the significance mask compared across conditions. ^***^P < 0.001.

The statistical test on the main effect Suppression revealed that the IC cluster localized near the left prefrontal cluster differentiated No Supp versus Supp (with or without Rwd) (Fig. 4). The location corresponds with previously reported DLPFC activation during eye-blink inhibition (Mazzone et al. 2010). The time–frequency analysis revealed theta-band (4–8 Hz) power increase for suppression conditions that started approximately −1.5 s prior to blink, which is a failure of blink suppression. These results may reflect increased effort to suppress blinks against increasing urge. Thus, we replicated anatomical location from the previous fMRI study, and furthermore succeeded in characterizing the modulation of brain dynamics as elevation of theta power during suppression with subsecond time resolution. The same comparison for the rest of the IC clusters are shown in Supplementary Material 6.

### Main Effect Urge

**Figure 5:**
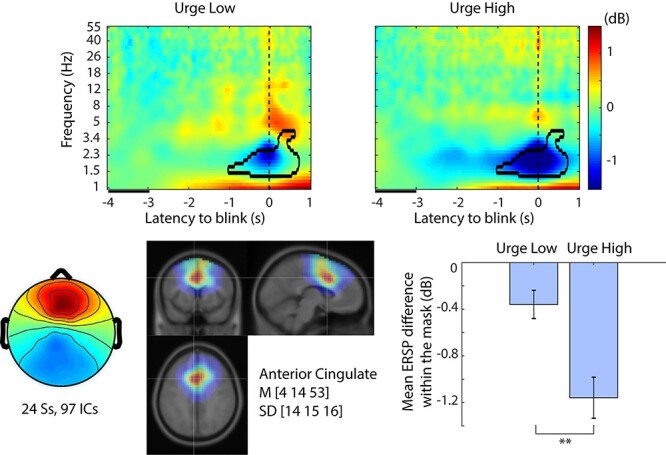
Event-related spectral perturbation (ERSP) plots for main effect Urge (Urge Low, Urge High) in the anterior cingulate independent component (IC) cluster. The contour mask in the time–frequency plots indicates *P* < 0.005 after controlling weak family-wise error rate (wFWER). Top row, ERSP for Urge Low and Urge High. Baseline period is indicated as a black line between −4 and −3 s relative to blink onset. Bottom left, cluster-mean IC scalp topography. Bottom center, cluster-mean dipole density with FWHM = 20 mm, and the centroid coordinate in the MNI template head. Bottom right, the mean ERSP values with SE within the significance mask compared across conditions. ^**^P < 0.005.

The statistical test on the main effect Urge revealed that the IC cluster localized near the anterior cingulate cluster differentiated Urge Low versus Urge High (Fig. 5). The location corresponds with a previously reported ACC activation during eye-blink inhibition (Mazzone et al. 2010). The time–frequency analysis revealed that subjective sense of urge was associated with power decrease in delta band (1–4 Hz) starting from 1 s prior to blinks. When we compare this delta-band ERSP suppression with the time-course of the urgeometer data for Urge High, we notice that the nonsignificant left tail of the delta-band suppression in Urge High starting from −3 s may be corresponding to a gradual increase of urgeometer values that started from −4 s. Also, the elbow point determined in the urgeometer data for Urge High (−1.8 s) seems to precede the ERSP difference (−1 s), but it is positioned in the middle of long left tail of this early nonsignificant portion of the continuum. Closer inspection of the significance mask indicates that the midpoint of the mask in time is not on zero but a few hundred milliseconds prior to zero, which may correspond to the fact that the urgometer peak was registered at −0.4 s. The significance threshold of *P* < 0.005 is arbitrary, and as such, exact agreement between the behavioral data and the EEG data in their time-courses may or may not occur; however, it is possible to see, in a general sense, the temporal correspondence between the urgeometer behavioral data and EEG modulations. The same comparison for the rest of the IC clusters is shown in Supplementary Material 7.

### Interaction Urge and Reward

**Figure 6:**
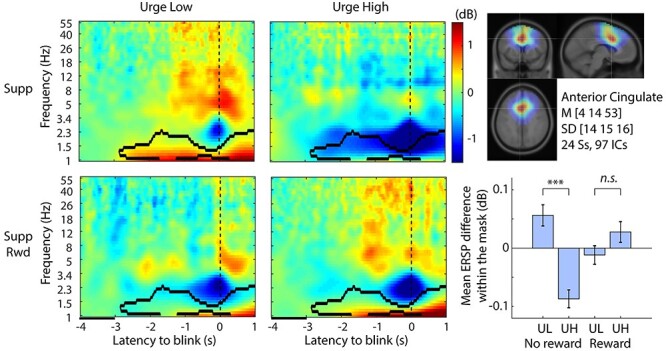
Event-related spectral perturbation (ERSP) plots for interaction Urge and Reward in the anterior cingulate independent component (IC) cluster. The contour mask in the time–frequency plots indicates *P* < 0.001 after controlling weak family-wise error rate (wFWER). Left 2 columns indicate ERSPs for the 2 × 2 conditions. Baseline period is indicated as a black line between −4 and −3 s relative to blink onset. Bottom left, cluster-mean IC scalp topography. Top right, cluster-mean dipole density with FWHM = 20 mm and the centroid coordinate in the MNI template head. Bottom right, the mean ERSP values with SE within the significance mask compared across conditions. ^***^P < 0.001. UL, Urge Low; UH, Urge High.

The same ACC cluster that showed the main effect of Urge reported above also showed significant interaction between Urge and Reward. While suppression of the delta band (1–4 Hz) power was associated with higher urge, the introduction of reward diminished this difference between Urge High and Urge Low; the results are shown in Figure 6. Interestingly, the significance masks from the urge (Fig. 5) and urge × reward (Fig. 6) do not overlap and the latency starts 2 s earlier in the latter analysis. This suggests that offering a Reward for successful suppression equalizes the response of the ACC region, regardless of urge intensity. It is also noteworthy that the significant interaction continued after blink onset, indicating that the ACC region may also be involved in postblink (i.e., suppression failure) processing, such as monitoring and evaluation. For interest, in order to minimize the effect of postblink brain dynamics, we truncated the mask at 0 s and performed the same statistics. The result still showed the same pattern as shown in Figure 6 bottom right, confirming that the obtained result is valid for the suppression period (data not shown). When using weak FWER correction, this operation violates the assumption of the cluster-level correction, so this test is limited to being a confirmatory process only.

## Discussion

In the current study, we asked 3 research questions: 1) How does urge develop? 2) What are the EEG correlates of blink suppression? 3) How does reward change brain dynamics related to urge suppression? Let us describe the answers to each of these questions: 1) There are at least 2 subtypes of urge development, Urge High and Urge Low. Urge High trials showed a well-defined waveform that starts to rise −5 s relative to blink, while Urge Low trials did not show much modulation; 2) Blink suppression was associated with EEG theta band power increase near or in the left dorsolateral prefrontal cortex (DLPFC); and 3) Reward suppressed urge-related EEG delta band power decrease near or in the anterior cingulate cortex (ACC). Below, we will discuss details and significance of the results.

Our results showed that trials grouped as Urge High showed a relatively slow time constant that started to rise from −5 s before a blink. At −1.8 s, the increase became steeper. At −0.4 s, the urge reached the peak. Around 2 s, it returned to baseline, and subsequently decreased below baseline (Fig. 2). In a prior fMRI study, a 1-min block-wise hemodynamic response model with linear increase toward the urge peak was used as a block-design regressor (Berman et al. 2012). However, this temporal model was designed heuristically and was not supported by empirical data. As far as we know, this is the first data to show the time course of building urge leading to a suppression failure. We also found nonlinearity of the urge increase, with which we may be able to model building urge more realistically. The result not only improves our understanding of urge time course but the temporal kernel we obtained in this study may be used in fMRI studies to estimate BOLD signal changes correlated with internal urge dynamics.

The separation of Urge High and Urge Low, defined by single-trial correlation to their mean value, was an ad-hoc decision as a part of data mining. The validity of this decision can be argued for 2 reasons. The first reason is that the relation between urge and suppression failure is not necessarily established. In a study of tic suppression, which is considered an analogue of blink suppression (van der Salm et al. 2018), it was reported that subjective ability to self-monitor urge increased with age (Banaschewski et al. 2003). This suggests that younger children may not have developed the ability to monitor urge, and failure of suppression may suddenly happen before becoming aware of the urge. Under this hypothetical uncertainty on reliability of self-report, separating single trials of suppression failures into subgroups of with and without self-report seems a valid first step to analyze the behavioral variance. The second reason is that the urgeometer time-series data for Urge Low and High indeed became separated into 2 distinguishable curves. The result plot seems to support the possibility that the (hidden) distribution of urge across single trials is rather binary, urge present or absent, than Gaussian. Note that the joystick we used may have had relatively small range of angle between neutral and maximum stick tilt, which could have made analogue resolution of the self-reported urge value limited. However, even if the input from the analogue joystick was effectively used as binary input, their statistical distribution across trials and participants should still be able to be studied as continuous probabilistic distribution. The rather binary urge distribution, which seems to have effectively 2 status, namely on and off, also seems to explain why taking a simple mean across all the trials is not a good idea here. Our exploratory blink ERP analysis also showed lower peak amplitude in blink ERP, suggesting that blink behavior could be different when urge levels are different. It leads us to speculate that blinks with low urge may be produced in more involuntary and reflexive way, hence they were faster and lighter than blinks with higher urge. Future studies on heterogeneity of single-trial self-reported urge expression with different age groups is awaited.

In the ERSP analysis, we focused on the preblink period during which blink suppression was still successfully maintained but about to collapse in a few seconds. The left prefrontal region showed distinctive EEG power increase prior to the blink during suppression conditions. The involvement of prefrontal regions (dorsolateral prefrontal cortex, DLPFC) in voluntary inhibition task has been reported repeatedly (Lerner et al. 2009; Mazzone et al. 2010; Aron et al. 2014). Our finding suggest that left prefrontal power increase is one of the EEG correlates of behavioral suppression, which is in line with these neuroimaging studies. Furthermore, our result provides rich time–frequency information. For example, we found this elevation started about −1.5 s to the blink with the present threshold. The urgeometer data showed that the urge increase rate changed at around −1.8 s, which seems to fit well with the ERSP time course. The data also showed that the EEG power increase was in the theta band (4–8 Hz), which suggests functional separation from, for example, the ACC region that showed EEG power decrease in the delta band (1–4 Hz) during the overlapping preblink period.

Analysis on main effect Urge revealed involvement of regions near anterior cingulate cortex (ACC), and Urge High was associated with deeper EEG power suppression compared with baseline period than Urge Low. ACC has been associated with various types of urges such as itch (Hsieh et al. 1994), voiding of urine (Kuhtz-Buschbeck et al. 2005; Griffiths et al. 2007), coughing (Mazzone et al. 2007; Leech et al. 2013), and smoking (Brody et al. 2004). Importantly, the same ACC cluster showed that subjective urge was modulated by availability of reward. This result was in line with our prediction that ACC is involved in subjective feeling, response coordination, self-monitoring, assessment of motivational valence, and initiation of motor actions (Medford and Critchley 2010). ACC has been known to be a region where regulatory and executive processes interact (Paus 2001). Involvement of ACC was also reported in a previous blink suppression study (Lerner et al. 2009) and antisaccade study (Milea et al. 2003). Not only did our scalp-recorded EEG results replicate these findings, our results showed for the first time subsecond temporal dynamics of how reward availability changes brain dynamics during subjective urge in the ACC region. The pattern of the interaction indicated that when reward is available, the urge-related ERSP power decrease was equalized between Urge Low and Urge High compared with the no reward condition. This may indicate that enhanced motivation by reward availability worked as a reinforcer of the top-down control over urge. This view seems to be in harmony with a network view of ACC together with insula, which we will discuss below.

ACC and insula are functionally closely related to each other. Both ACC and insula commonly contain von Economo neurons (Allman et al. 2010), which are large bipolar neurons that are unique to these regions and also unique to great apes and humans. There is anatomical evidence that ACC, specifically Brodmann area 24 here, has reciprocal connection with insular cortex (Mesulam and Mufson 1982; Vogt and Pandya 1987), and this connection may be mediated by von Economo neurons (Craig 2009). Thanks to this reciprocity, not only does insula integrate sensory information to generate awareness, which is then transferred to ACC for evaluating with various other information, making decisions, and initiating motor commands (feedforward connection), but also the result of the processing in ACC may be back-projected to insula to modulate how subjective awareness is formed (Medford and Critchley 2010) (feedback connection). This view seems to be supported by empirical evidence that the placebo effect for antitussive therapies is generally substantial, but it turned out to be associated with modulation in activation of a cortical network including ACC and insula (Leech et al. 2013). The result indicates that the ACC-insula network was one of the major brain regions that received modulation just by top-down belief that lead to change in behavior. We speculate that reward availability in our study might be related to the same network, and ACC may have played a critical role in changing the behavior of blink suppression when reward was available.

The current finding may have clinical value for, for example, designing a behavioral training program for children with Tourette’s syndrome (Woods and Himle 2004; Greene et al. 2015). Our results provided evidence of neural substrata underlying the behavioral suppression. Together with other literature of neuroimaging studies, our results can provide spatio-temporally resolved neural mechanism of behavioral suppression. Particularly, the parallel time courses of behavioral and electrophysiological dynamics toward suppression failure we showed in this study seem capable of providing a spatio-temporal target for treatment using transcranial magnetic stimulation (TMS) for tic patients. Future studies toward this direction is awaited.

### Limitation

The results of this study are the first of their kind and as such should be considered preliminary until independent replication occurs. Additional limitations are noted as follows. The presence of blink and related muscle artifact in EEG recording typically creates critical limitation. We addressed this issue by using 2 approaches. One of the approaches was to set the main time window of analysis prior to the blink onset. In fact, eye blink in this study indicates the offset of the time window of interest. The other approach was to employ independent component (IC) modeling approach (Onton and Makeig 2006) rather than scalp electrode signal analysis. We performed a post hoc simulation study, which can be found in Supplementary Material 5.

The use of the urgeometer in the current study can be argued. The ability to self-monitor urge depends on age (Banaschewski et al. 2003). In addition, using the urgeometer may impose multitasking of self-monitoring and motor execution, which may have interfered with suppression performance and associated brain dynamics. Care needs to be taken when we interpret the ERSP differences between No Supp (no urgeometer use) and other Supp conditions (urgeometer used).

We relied on suppression-breaking blinks to define the suppression period. For this reason, we needed to exclude several participants with fewer number of blinks. It may seem possible to define “successful suppression” when urgeometer showed a high value but no blink followed. But this approach has 2 problems: 1) we do not know whether urge could disappear with continued suppression and 2) an additional condition is necessary to consider in which the urgeometer is used irrespective of urge to counterbalance the brain dynamics related to motor planning and execution. Future studies with the suggested conditions may be helpful to validate the use of the urgeometer.

Due to known dependencies of scalp EEG recording on cytoarchitecture, source distance and geometry (Nunez and Srinivasan 2006), our main results were limited to cortical sources close to the surface, and contribution of deeper sources such as insula was not detected. Moreover, in the case of insula, it is also reported that active source area spreads rapidly to the surrounding structures (Sun et al. 2015), which makes it difficult to form a temporally stable active cortical patch that can be detected at scalp recording. In epilepsy studies, differentiation of insular seizures from temporal, parietal, and frontal lobe seizures (Isnard et al. 2004; Nguyen et al. 2009) was not possible, suggesting that both interictal and ictal recordings might fail to display epileptiform discharges for insular seizures (Desai et al. 2011; Ryvlin and Picard 2017). Thus, it is generally hard to obtain insular activity with scalp EEG recording. For interpreting the current results, however, there are a large number of anatomical and neuroimaging studies on the insula and ACC-insula network. As we demonstrated above, using the wealth of literature to interpolate the lack of insular and basal contributions seems necessary to interpret the current EEG result in the context of neuroimaging studies on blink suppression.

## Conclusion

We demonstrated that 1) blink suppression was associated with EEG theta band power increase near or in the left DLPFC; 2) reward improved suppression performance, and Reward suppressed urge-related EEG delta band power decrease near or in the ACC; and 3) real-time self-reported urge has single-peaked time-course longer than 7 s (and peaking at −.4 s), but this applies only to half of failed suppression trials.

## Supplementary Material

Supplementary_Material_revision1_tgaa046
