# Supplementary material for: Modulation of Frontal Oscillatory Power during Blink Suppression in Children: Effects of Premonitory Urge and Reward
Source: Cereb Cortex Commun. 2020 Aug 6;1(1):tgaa046. doi: 10.1093/texcom/tgaa046 (PMC8153050; doi:10.1093/texcom/tgaa046)
Supplement: Supplementary_Material_revision1_tgaa046 [file supplementary_material_revision1_tgaa046.docx]

Supplementary Material

**Modulation of frontal oscillatory power during blink suppression in children: Effects of premonitory urge and reward**

Makoto Miyakoshi^a*^, Joseph Jurgiel^b^, Andrea Dillon^b^, Susanna Chang^b^, John Piacentini^b^, Scott Makeig^a^, Sandra K. Loo^b^

^a^Swartz Center for Neural Computation, Institute for Neural Computation, University of California San Diego, 9500 Gilman Drive La Jolla CA 92093-0559

^b^Semel Institute for Neuroscience and Human Behavior, University of California, Los Angeles, 760 Westwood Plaza, Los Angeles, CA 90095

*Corresponding author:

Makoto Miyakoshi, Ph.D.

Swartz Center for Neural Computation, Institute for Neural Computation, University of California San Diego

9500 Gilman Drive La Jolla CA 92093-0559 USA

Office Phone: 858-825-7534

Email: mmiyakoshi@ucsd.edu

Contents

[1. EEG Preprocessing 2](#_Toc44756119)

[2. Influence of Eye-IC on Scalp Sensor Data 3](#_Toc44756120)

[3. Main effect of Suppression and Urge on EOG 5](#_Toc44756121)

[4. Histogram of blink occurrence. 6](#_Toc44756122)

[5. Post-hoc Validation of EEG Preprocessing 7](#_Toc44756123)

[6. Description of Artifact Subspace Reconstruction (ASR) 13](#_Toc44756124)

[7. ERSP results for all clusters, all conditions. 15](#_Toc44756125)

[8. Bibliography 18](#_Toc44756126)

# EEG Preprocessing

We show the step-by-step processes in order below.

1. EEG data were imported from the original recording format to EEGLAB. The digitized channels locations were imported. The scalp channel time-series data were downsampled to 250 Hz.
2. Data were high-pass filtered with cutoff frequency at 1.5 Hz (FIR, Blackman window, transition bandwidth 1.0Hz, filter order 1376) and low-pass filtered at 55 Hz (FIR, Blackman window, transition bandwidth 10Hz, filter order 138). The relatively high cutoff frequency for high-pass filter is to enforce stationarity of the data to facilitate ICA (Winkler et al. 2015). The low-pass filter is to suppress the line noise at 60 Hz.
3. clean_rawdata() plugin including ASR was applied with the following parameters: channel rejection for poor correlation with surrounding channels, 0.75; ASR with correction criterion in SD, 8; and the final window rejection for poor data quality, 0.5. See Supplementary Material 5 for detail of the algorithm used in ASR.
4. Spline interpolation was applied to the rejected channels. This is to compute an unbiased average-reference potential in the next step.
5. Average reference was applied to the data. In doing so, the original reference channel, which is continuous zeros, was included so that the averaging did not change data rank.
6. Single-model adaptive mixture ICA (AMICA) (Palmer et al. 2008, 2016; Delorme et al. 2012; Hsu et al. 2018) was performed decomposition with the following parameters: SD for datapoint rejection, 3; number of rejection, 15. This set of parameter rejects around 10% of data points only for the AMICA purpose.
7. Equivalent current dipoles were fit using Fieldtrip (Oostenveld et al. 2011) for scalp projections of the independent components. Symmetric two dipoles were fitted where necessary (Piazza et al. 2016).
8. EEGLAB plugin ICLabel() was used to generate probabilistic labels such as ‘Brain’, ‘Eye’, ‘Muscle’ for each IC (Pion-Tonachini et al. 2019) to evaluate impact of ICA-decomposed eye component source activations on scalp sensor signals.
9. Independent component selection was performed in the following way. Using EEGLAB STUDY, all subjects’ ICs whose equivalent dipole locations were inside the MNI brain mask and have less than 15% residual variance (i.e., comparison between the ideal dipolar projection on the scalp and the empirically obtained scalp topography of ICs) were classified into 30 clusters using k-means algorithm on IC’s precomputed power spectral density (PSD). Clusters with non-brain PSDs were selected for rejection. By backprojecting the remaining IC clusters, individual data were reconstructed. All of these processed were performed using EEGLAB plugin *std_clust2ch()*.
10. Using the reconstructed data, individual data were processed for the final group-level clustering analysis. First, the continuous data were epoched into +/- 5.7 s windows relative to blink ERP peak. The epochs were labeled for ‘No Supp’ ‘Supp’ ‘Supp Rwd’ according to the block-separated conditions they belong to. Then, EEGLAB STUDY was built again for the final statistics. For each subject and condition, event-related spectral perturbation (ERSP) (Makeig 1993) was computed using the following parameters: Frequency range, 1 to 55 Hz, number of wavelet cycles, from 3 to 15 (linear increase), sliding window width, 3340 ms, number of output times, 403 (so that the mean window step size is about 20 ms starting from -4030 to 4026 ms), number of frequency bins, 100. Baseline period was defined from -4000 to -3000 ms (see Supplementary Material 3 for the rationale for this decision). K-means clustering was performed using IC dipole locations only without any time-frequency data to avoid circular inference in the subsequent statistical tests (Kriegeskorte et al. 2009). The number of clusters was determined by using optimization algorithms including Silhouette (Rousseeuw 1987), Davies-Bouldin (Davies and Bouldin 1979), and Calinski-Harabasz (Calinski and Harabasz 1974) to see if their suggestion would converge. We adopted the minimum number suggested to maximize the unique subject ratio per IC cluster.

# Influence of Eye-IC on Scalp Sensor Data

To evaluate the influence of blink artifact on scalp sensor data, we examined group-level ERPs that were averaged across the eight front most channels located around the forehead (see Figure S1). The ERPs were time-locked to single-trial EOG peak latency. We compared the ERPs across the following three conditions: 1) minimally cleaned sensor data (only outliers were removed); 2) re-constructed scalp sensor data by back-projecting 180/4464 ICs (from all 35 participants) labeled as ‘Eye’ with > 0.50 probability, 3) re-constructed scalp sensor data by back-projecting 910/3224 ICs (from the finally selected 26 participants) that were qualified brain components submitted for the final group-level analysis. The results showed that the back-projection of 180 eye ICs modeled the high-amplitude EOG almost completely (Figure S1). This indicated that ICA successfully captured nearly the entire variance of EOG artifact with as little as 4% of the total number of ICs, and the remaining ICs are maximally independent (i.e. free of) this ocular artifact. To demonstrate the scale difference between the backprojections of decomposed EOG components and the finally selected qualified brain components, ERP of the latter was overlaid on Figure S1.


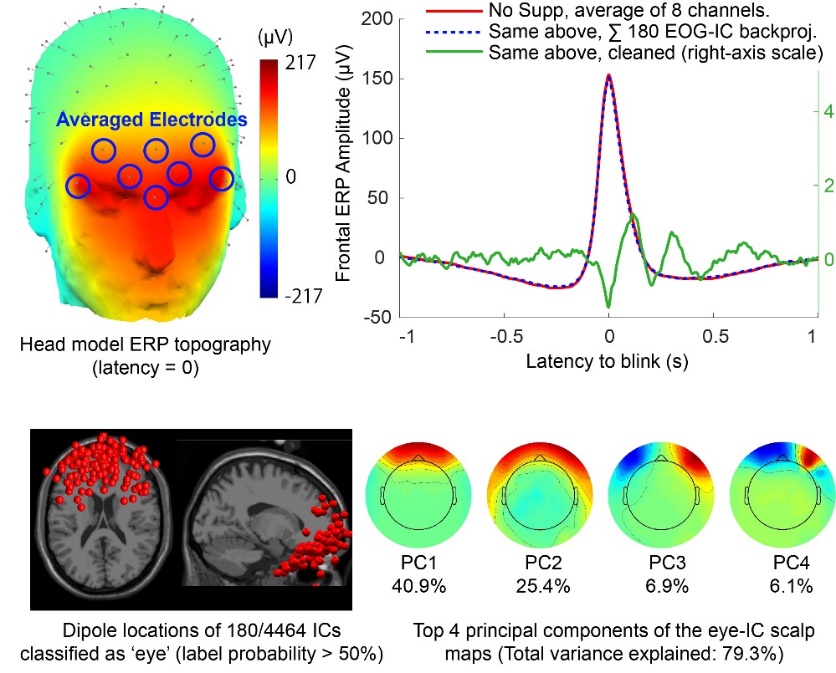


*Figure S1. Top left, the head topography of blink ERP at the peak latency. The front most scalp electrode locations were shown. The ERPs recorded at these electrodes were averaged to serve as representative blink ERP. Top right, the comparison across minimally processed raw blink ERP, the scalp back projection of 180/4464 ‘eye’ independent components (ICs) identified by ICLabel algorithm (Pion-Tonachini et al. 2019) with label probability > 50%, and the scalp back projection of 910/3224 qualified brain ICs used for the final analysis. Note the difference in the two amplitude scales shown in the left and the right vertical axes. Bottom left, the equivalent current dipole locations of the ‘eye’ ICs. Bottom right, mean scalp topographies of top 4 principal components (PCs) obtained from the scalp topographies of ‘eye’ ICs. PC1 and PC2 correspond to blink and/or vertical eye movement, while PC3 and PC4 horizontal eye movement.*

# Main effect of Suppression and Urge on EOG

Next, we tested whether blink behavior, characterized by ERPs time-locked to the EOG peak, was affected by Suppression and/or Urge. The ERPs of the minimally cleaned data averaged across the eight forehead sensors tested in Figure S1 were compared. The results did not show statistical differences across Suppression conditions (Figure S2). However, when trials were separated into High and Low Urge conditions, the difference between the two EOG peak amplitudes reached significance when uncorrected for multiple comparisons. Although the uncorrected statistical result requires caution for interpretation, the smaller EOG peak for Urge Low may indicate that when subjective urge is low (or unmonitorable), the corresponding blink may be physically smaller than High Urge blinks.


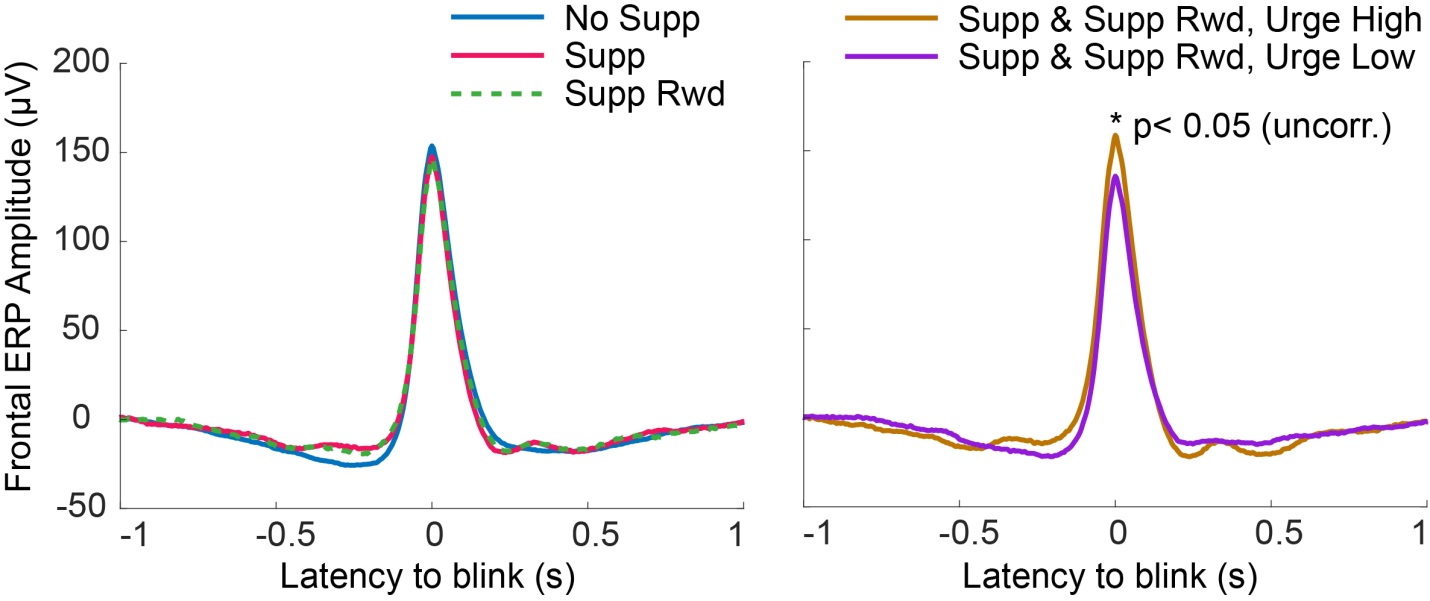


*Figure S2. Blink ERPs separated into experimental conditions. Left, the condition Suppression was tested. The peak ERP amplitude did not show statistical difference. Right, the condition Urge was tested. The peak ERP amplitude showed statistical difference at an uncorrected p-value.*

*Scalp-ERP Comparison Between Before and After Data Cleaning*

To study the effect of artifact removal, we compared scalp sensor-level 128 ch ERPs before and after the data cleaning processes (Figure S3). It is clear that the blink ERP has high amplitude and broad distribution along with the anterior-posterior axis of the head. It is notable that after artifact rejection, the amplitude scale was nearly 25 times reduced. Also, the anterior-posterior distribution of the ERP did not show a classical, well-defined waveform. The visual impression motivated us to use time-frequency decomposition as the main analysis approach rather than quantifying ERP waveforms.


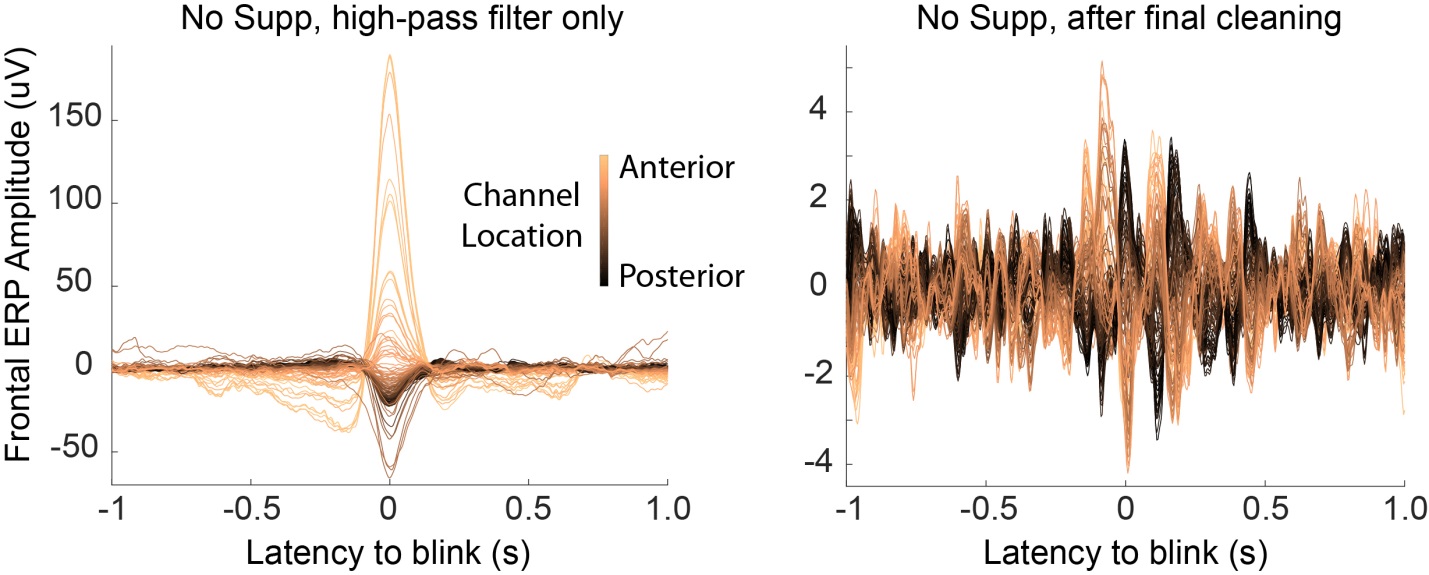


*Figure S3. The grand-mean ERP butterfly plots of 128 scalp sensor recordings. The plotting color scale represents y axis coordinates of the electrodes on the template MNI head. Left, minimally processed ERP data. Right, after final cleaning. Note the amplitude scale difference between the two plots (200 μV vs. 8 μV). One may be concerned about the drastic amplitude reduction and wonder if targeted brain signals still remain. This question is answered in Extended Data 4 by a post-hoc validation test.*

# Histogram of blink occurrence.

**
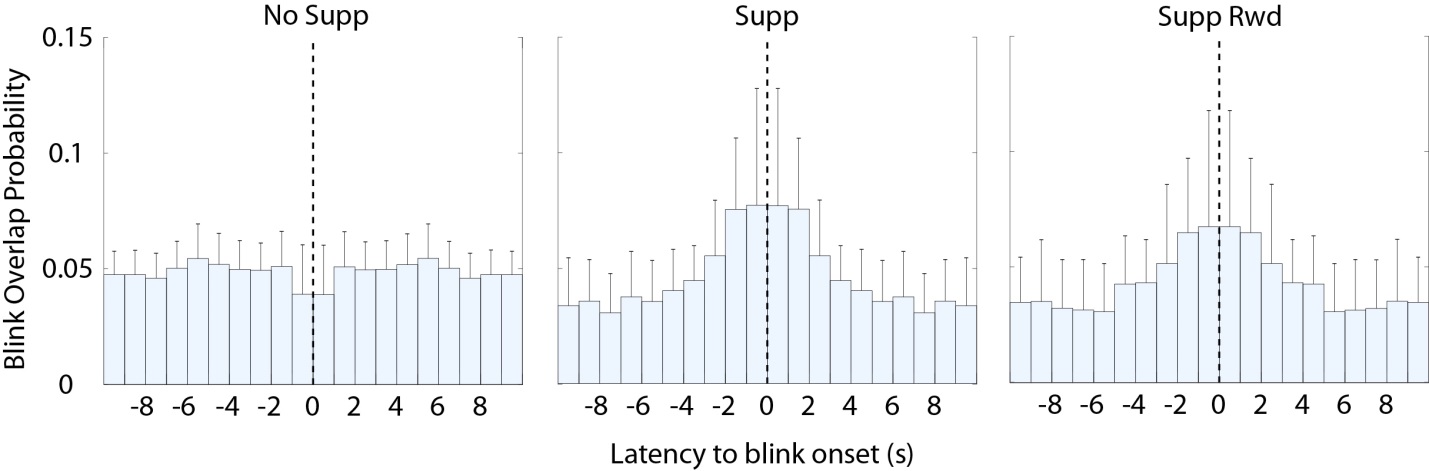
**

*Figure S4. Histogram of blink probability relative to a blink ERP peak. The rise toward latency zero started at around -3 s. Based on this evaluation, we determined to set the baseline period in our event-related spectral perturbation to be -4 to -3 s.*

# Post-hoc Validation of EEG Preprocessing

In the previous section, we reported that mean of 99.7% of variance was reduced from the raw data. This number may appear surprisingly large if one is not familiar with these data cleaning statistics, and may wonder if EEG signal is still present after the cleaning. This is a legitimate and critical question that concerns validity of data presented here.

To answer this question properly, ideally we would need a study with a known signal embedded as ground truth to calculate change of signal-to-noise ratio (SNR) between before and after the cleaning process. However, such a ground truth signal is usually unavailable for a standard human EEG study. The present study does not include such a design for hard validation either. We cannot obtain analytic solution to determine the effect of data cleaning process either, since results from ASR and ICA are data dependent. However, it seems still possible to perform a post-hoc validation to provide, at least, some type of lower bound of confidence interval of the current cleaning method and insight for quantitative characteristics relevant to it. This validation, however, critically depends on assumptions of ICA and how it works. The schematic illustration for the procedure of the post-hoc validation is shown in Figure S5.


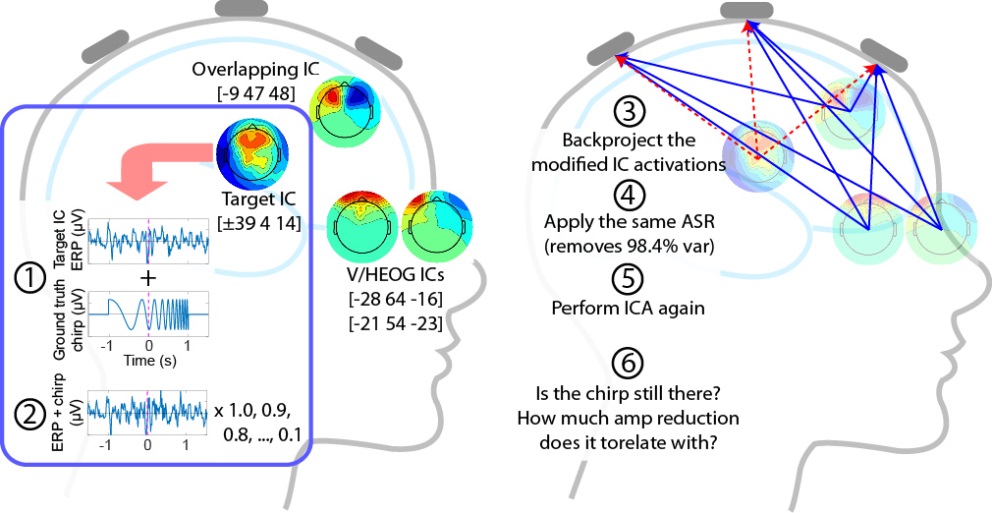


*Figure S5. Schematic illustration of the procedure used in the post-hoc validation.*

Below, we describe the procedure step by step.

1. A single subject from whom we obtained the least number of the ICs (10) for the final analysis was chosen for this validation. This is to assume the worst case scenario where data were most challenged in terms of quality of ICA results among all the subjects. We gave additional and more aggressive data cleaning based on manual data-window rejection on this dataset only for the purpose of the current post-hoc validation study and ran the second ICA so that the definition of ‘clean EEG’ and the associated ICA weight matrix is best maintained. From the second ICA, we obtained 14 ICs classified as ‘brain’ with > 0.8 probability by *IClabel()* plugin (Pion-Tonachini et al., 2019). We copied this ICA weight matrix to the same subject’s raw EEG data (after rejecting the same channels rejected for the ICA 116 ch, the same referencing to average, and applying the same high-pass filter). Thus, the raw scalp EEG data were decomposed with ICA calculated from the aggressively cleaned data, though the artifacts rejected by ASR were still fully present. We selected one of the ‘brain’-labeled ICs (probability: 0.90) that was localized in the frontal lobe whose Talailach coordinate [+/-39 4 14]; symmetrically bilateral dipoles were fit suggested by *twoDipoleFit()* plugin (Piazza et al. 2016). Henceforth this IC will be referred to as *target IC*. Also, in order to evaluate potential leakage from the target IC due to crosstalk between ICs by imperfectly achieved temporal independence, we selected another IC localized at [-7 59 36] whose scalp projection showed overlap with that of *target IC*. Henceforth, this IC will be referred to as *overlapping IC*. Finally, we selected vertical EOG/blink and horizontal EOG ICs as *eye ICs* (label probability: 0.86 and 0.99). We calculated variance of the *target IC* and the *eye ICs* between -1 to 1 s relative to blink ERP peak to calculate signal-to-noise ratio (SNR), which is defined as 20*log10(var(*target IC*)/var(*eye ICs*)) in terms of representative ocular artifacts. The SNR for the horizontal and vertical/blink *eye ICs* were averaged into a single value.
2. A chirp signal (log-scale increase from 10 to 50 Hz, -1 to 1 s relative to blink ERP peak) was added to the single-trial ERP of *target IC.* This chirp signal was scaled to have only 1% of standard deviation of the corresponding raw signal (7.05 vs. 0.07) so that it impacts performance of ICA minimally (see quantitative comparison below). This low-amplitude short-burst chirp signal served as a ground truth, representing a pre-defined time-frequency characteristics that is time-locked to blink ERP peak. Care was taken not to affect the statistical characteristics of the time-series data of the *target IC*: The comparison before and after adding the chirp signal showed that standard deviation was 7.0461 vs. 7.0466 (0.07% change), skewness was -1.7569 vs. -1.7564 (0.03% change), and kurtosis was 52.4106 vs. 52.3946 (0.03% change), respectively. Overall, the effect of adding the chirp to the original signal was < 0.1%, which was expected to have only negligible influence in the subsequent ICA.
3. The IC activation time series, including the embedded chirp signal in the *target IC* activations, was backprojected to scalp sensors. In this way, the scalp sensor data were reconstructed with the ground truth signal during the peri-blink period. Importantly, this backprojection was performed 10 times while the amplitude of the *target IC* activations was reduced from 100% to 10% with 10% step. Henceforth, the backprojected (BP) data with reduced *target IC* amplitude will be referred to as BPtargetIC_100%, BPtargetIC_90%, BPtargetIC_80%, BPtargetIC_70%, …, BPtargetIC_10%,  and the original unmodified ICA result as BP_original_.
4. The backprojected scalp channel data were processed with the ASR with the same parameters used in the main study.
5. ICA was performed with the same parameters used in the main study.
6. The decomposed ground truth signal after various levels of SNR reduction was evaluated to determine how much SNR loss can be tolerated by the current cleaning method (ASR + ICA), thus to estimate the lower bound of confidence interval and margin for the current data in the worst case scenario.

The results are plotted Table S1 and Figure S6. Before and after ASR, SNR gain of 3.89 dB was confirmed. As the amplitude scale of the *target IC* was reduced progressively, the SNR decreased in relatively linearly. However, IC scalp topography correlation coefficient showed a clear knee point at amplitude scaling to 40%, which was determined by finding a bisection point that minimizes the sum of errors for the two linear line fits. The SNR difference between 100% and 40% amplitude scaling points was 0.63 - (-6.34) = 6.97 dB. This amount of SNR may be interpreted as a margin from the lower bound of confidence interval of the analysis used in the current study.

*Table S1. The target IC statistics for BP_original_, BPtargetIC_100% to BPtargetIC_10%.*

|  | Orig. | 100% | 90% | 80% | 70% | 60% | 50% | 40% | 30% | 20% | 10% |
| --- | --- | --- | --- | --- | --- | --- | --- | --- | --- | --- | --- |
| Target IC variance | 4.560 | 4.656 | 3.811 | 3.149 | 2.456 | 1.964 | 1.499 | 0.978 | 0.693 | 0.517 | 0.241 |
| Variance rank | 3 | 3 | 5 | 6 | 8 | 8 | 11 | 16 | 19 | 21 | 40 |
| Scalp topo corr. | 1.000 | 0.995 | 0.999 | 0.991 | 0.973 | 0.967 | 0.938 | 0.934 | 0.876 | 0.741 | 0.639 |


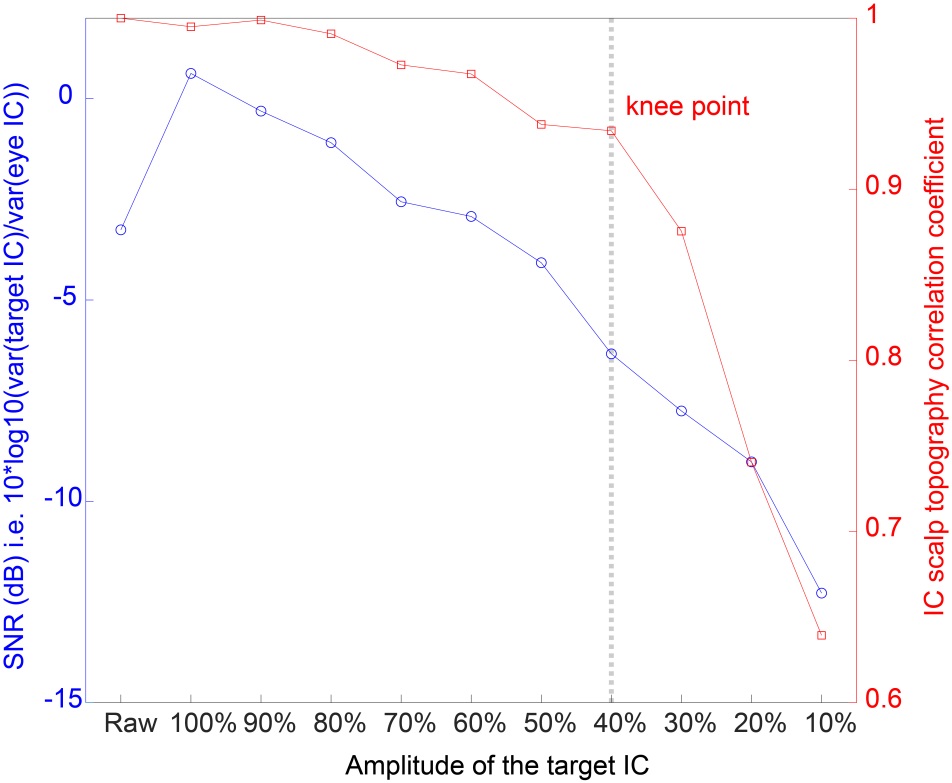


*Figure S6. Changes of SNR, defined by target IC variance over eye IC variance, and IC scalp topography correlation with the original ICA decomposition along with amplitude scale reduction of the target IC. The knee point defined by bisection point by two line fitting that minimizes error was found at 40%, suggesting that the ASR+ICA method used in the current study has margin of 6.97 dB in SNR.*

Next, we evaluated how the implemented ground truth survived the ASR + ICA cleaning processes. The results are shown in Figure S7. The target IC did not show visually identifiable modulation in ERSP. However, inter-trial phase coherence (ITC) showed the time-frequency signature of the ground-truth chirp signal surprisingly robustly down to the scale reduction to 10% (SNR loss of 12.90 dB). The result indicates that the current ASR+ICA approach can detect weak signal robustly (as long as they fit the assumption of ICA) and does not critically affect time-frequency characteristics.


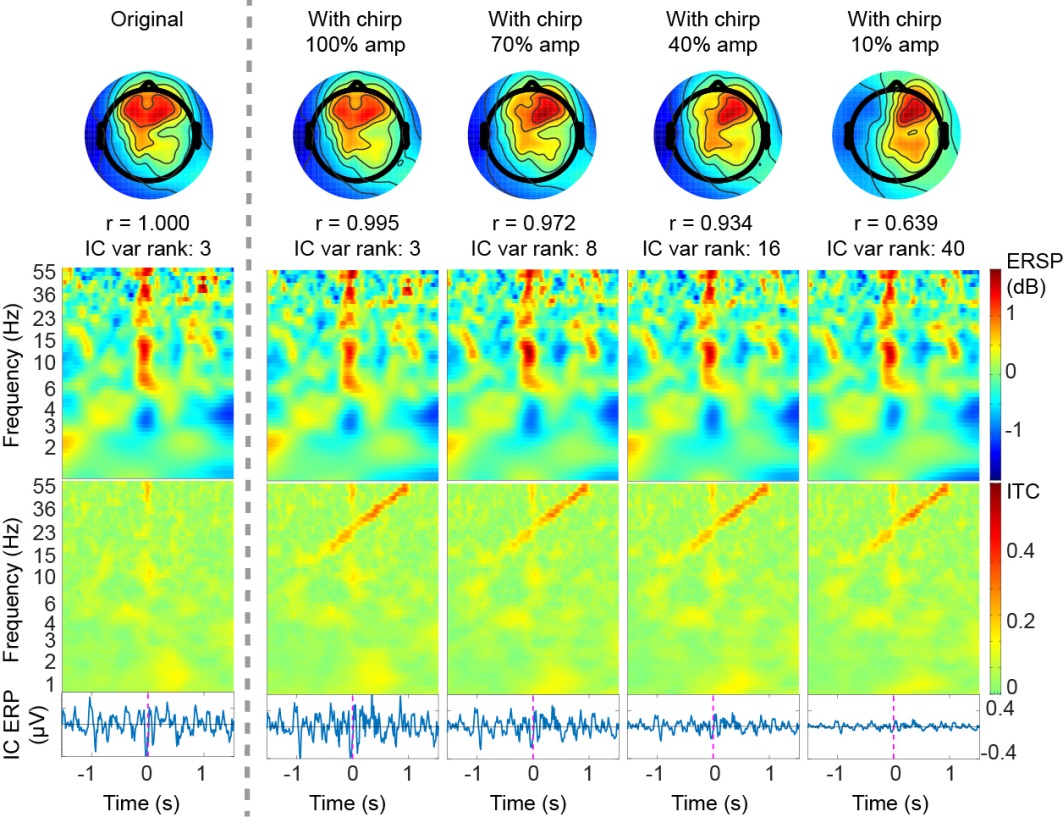


*Figure S7. Time-frequency analysis on the target IC during peri-blink period. Event-related spectral perturbation (ERSP, the second row) did not show the signature of chirp, but inter-trial phase coherence (ITC, the third row) detected the chirp pattern down to 10% amplitude scale. ERSP was calculated as a power ratio against mean power value during the baseline period, hence it remained unaffected by the general amplitude reduction. The IC ERP waveforms (the bottom row) showed progressively diminishing amplitude. The result demonstrated that the small-amplitude (1% variance of the component) ground truth signal survived ASR+ICA data cleaning method used in this study even when the component amplitude was reduced to 10 % (SNR loss of 12.90 dB).*

Finally, we evaluated potential leak or crosstalk effect due to insufficient decomposition between *target IC* and *overlapping IC* as the amplitude scale for the *target IC* was reduced. This test may be understood as testing specificity, in contrast with sensitivity shown in Figure S8. Since we confirmed ERSP did not show noticeable evidence of the presence of the ground-truth chirp signal, we focused only on ITC differences from the original ITC results. The results are shown in Table S2 and Figure S8. The characteristic time-frequency pattern of the chirp became noticeable as the amplitude downscaling on the *target IC* progressed, indicating potential leak or crosstalk between ICs was confirmed. Note that 70% condition also showed noticeable chirp (data not shown), indicating the leak/crosstalk effect is nonlinear.

*Table S2. The overlapping IC statistics for BP_original_, BPtargetIC_100% to BPtargetIC_10%*

|  | Orig. | 100% | 90% | 80% | 70% | 60% | 50% | 40% | 30% | 20% | 10% |
| --- | --- | --- | --- | --- | --- | --- | --- | --- | --- | --- | --- |
| Overlap. IC var. | 1.478 | 1.515 | 1.527 | 1.461 | 1.534 | 1.516 | 1.513 | 1.420 | 1.479 | 1.458 | 1.508­­­­­­­­­­­­ |
| Variance rank | 12 | 12 | 12 | 12 | 12 | 12 | 12 | 11 | 11 | 11 | 11 |
| Scalp topo corr. | 1.000 | 0.999 | 0.999 | 0.987 | 0.908 | 0.986 | 0.987 | 0.983 | 0.972 | 0.965 | 0.983 |


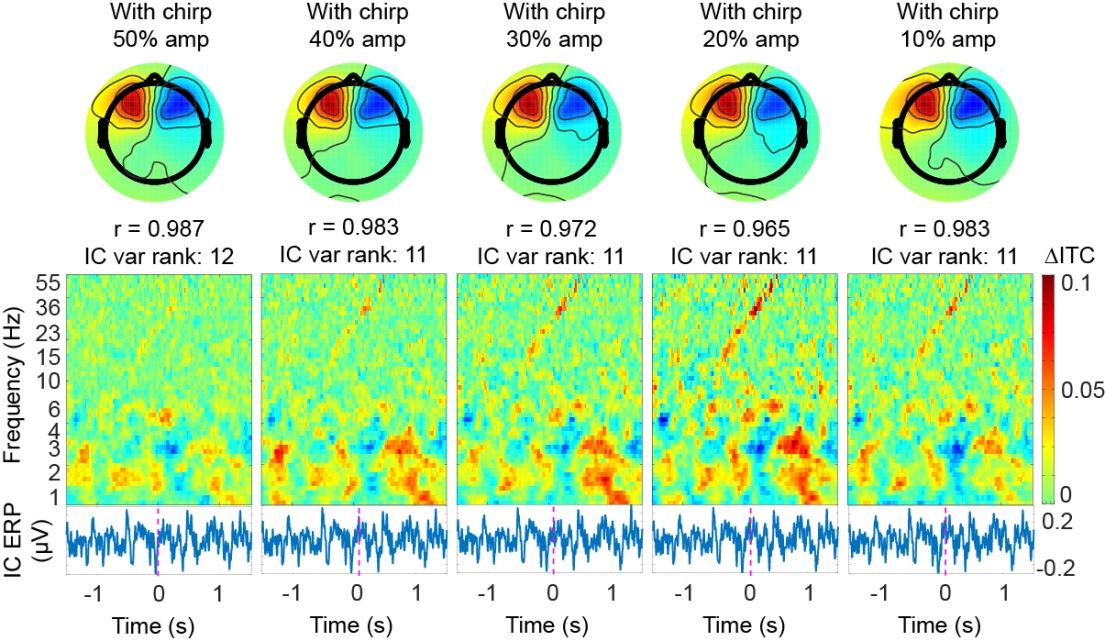


*Figure S8. Time-frequency analysis on the overlapping IC during peri-blink period. ITC differences (i.e., subtractions) from the original data ITC are shown. Because target IC and overlapping IC are by definition temporally maximally independent, the characteristic time-frequency pattern of the ground-truth chirp signal should not be present in the overlapping IC. However, due to the progressive SNR reduction by reducing the target IC amplitude, ICA started to fail to keep the ground truth signal only to the target IC. This may be understood as leakage or crosstalk between ICs under progressively challenging situation in terms of SNR. Note the difference of color scale used in Figure S7 (0.6 vs. 0.1) which indicates minor magnitude of this phenomenon.*

In summary, we attempted to answer to the legitimate skepticism that such a heavy artifact rejection preprocess that removes more than 99% of data variance may also lose signal of interest altogether. The results from the post-hoc validation tests confirmed that 1) ASR alone showed 3.89 dB of SNR gain; 2) deterioration of IC scalp projection was observed when signal variance was reduced to less than 40%; 3) Time-frequency characteristics of the ground truth signal survived ASR + ICA processes even with 12.90 dB of SNR loss (evaluation of sensitivity); 4) Inter-IC leakages or cross-talk started to happen when signal variance was reduced to less than 40% (evaluation of independence). We concluded that target single can tolerate with variance reduction down to 40%, which is SNR reduction of 6.97 dB. This value indicates SNR margin in this example scenario, which can be understood as lower bound of confidence interval of the entire processing methods we used in the current study. Among similar studies, such validation is rarely shown. We hope our attempt here showed the level of reliability we expect to our data, and demonstrated an idea of performing quantitative evaluation of ASR-ICA approach, which can be further extended to be a separate methodological development by taking advantage of data decomposition using ICA.

# Description of Artifact Subspace Reconstruction (ASR)

In the conventional scalp EEG studies, it was a widely believed that that EEG recording during blink must be rejected (Picton et al. 2000). A common practice to perform this is to use an amplitude threshold for a rejection criterion, and representative value could be anywhere between +/-75 and 200 μV. However, our question was to investigate brain dynamics related to blink. To analyze such data, we used two artifact rejection approaches, namely artifact subspace reconstruction (ASR) and independent component analysis (ICA) to address the issue of stationary and non-stationary artifact, respectively. A historical fact is that the offline version of artifact subspace reconstruction (ASR) implemented in *clean_rawdata()* plugin, which is now validated by multiple studies (Mullen et al. 2015; Chang et al. 2018, 2019; Gabard-Durnam et al. 2018; Blum et al. 2019; Plechawska-Wojcik et al. 2019), was specifically developed for this project upon our request by the main developer of BCILAB (Kothe and Makeig 2013). The original solution was called *Christian-Nima Combo* after the developers, but formally changed into *clean_rawdata()* on June 26, 2013 to be implemented as an plugin for EEGLAB (Delorme and Makeig 2004). The detail of the algorithm is described below. Importantly, ASR works in a complementary way with ICA; ASR uses sliding-window interpolation (to be exact, subspace reconstruction using principal component analysis) to correct non-stationary artifacts that ICA cannot handle. This solution improves data stationarity, which is a critical assumption for ICA that practically works as a requirement to obtain good performance.

Artifact subspace reconstruction (ASR) removes short-duration, high-amplitude artifacts in the continuous data using 0.5-s sliding window with 50% overlap and linear blending. The detailed flow of the process is explained here. Let $X_{calibrate}\in\Re^{Q\times M}$ be ‘the cleanest part of the channel data’ which ASR finds for calibration for Q-channel continuous EEG data. This is achieved by first applying infinite impulse response (IIR) spectral weighting (8^th^ order Yule-Walker) that is the inverse of an in-house developed heuristic model of EEG power spectral density that has 1/f curve with an attenuated lower-frequency end and a peak at around 8 Hz, then concatenating windows of time-series by z-scored RMS amplitude of the time series between -3.5 and 5.5 after. Let $\bar{M}$ the square root of the covariance matrix $C$of $X_{calibrate}$ such that where $\bar{M}^{T}$ represents transpose of $\bar{M}$. Let $x_{(t)}\in\Re^{Q}$be a EEG sample at time point $t$, and let $X\in\Re^{Q\times N}$be the sliding window of data containing $x_{(t)}$. Principal component (PC) analysis (PCA) is applied to $X$ to obtain PCs of the currently selected window $V=\left[v_{1}...v_{Q}\right]\in\Re^{Q\times Q}$. Those PCs $V$ whose variance $d_{k}$ exceeds an omnibus cutoff threshold $h(v_{k})$are determined as *artifact subspace*. The omnibus cutoff threshold $h_{k}$ is defined as follows. Let $M=V^{T}\bar{M}$be projection of $\bar{M}$into PC space. Principal component analysis on $X_{calibrate}$ is computed to obtain $W=\left[w_{1}...w_{Q}\right]\in\Re^{QxQ}$ and activations $Y=W^{T}X_{calibrate}$. For activation of each component $Y_{k}$, root-mean-square amplitude is calculated to obtain robust mean $\mu_{calibrate}$ and standard deviation $\sigma_{calibrate}$. The omnibus threshold is obtained as $h_{k}=\mu_{calibrate\_k}+\sigma_{calibrate\_k}\times f_{user}$ where $f_{user}$ represents user-specified factor for SD. For the current analysis, we chose $f_{user}=8$ which is a very lax criterion, intending only to remove large outlier activations in the time-series data in order to let the subsequent ICA decompose artifacts that have physiological sources, such as eye blinks/movements, EMG, and ECG.

After artifact subspace is determined, the threshold operator $U\in\Re^{Q\times Q}$ is built. For this, the omnibus threshold $h_{k}$ in the channel space needs to be projected to the PC space $h(v_{j})$ as $h(v_{j})=\sum_{k} \left[(\mu_{calibrate\_k}+\sigma_{calibrate\_k}\times f_{user})W_{k}^{T}V_{j}\right]^{2}$ to be compared with $d_{j}$. Now, $U_{jl}$ is built such that $U_{jl}=0$ if $d_{j}>h(v_{j})$ to remove them, otherwise $U_{jl}=1$ to keep them. After removing artifact subspace, it is interpolated, which completes the process *artifact subspace reconstruction.* This process is represented by the linear operator $R=VM(M\circ U)^{+}V^{T}$ such that $\hat{x}_{(t)}=Rx_{(t)}$ produces corrected data $\hat{x}_{(t)}$for input data $x_{(t)}$. $M\circ U$represents element-wise multiplication of $M$ and $U$, and $(M\circ U)^{+}$ represents Moore-Penrose pseudoinverse of $(M\circ U)$.

# ERSP results for all clusters, all conditions.


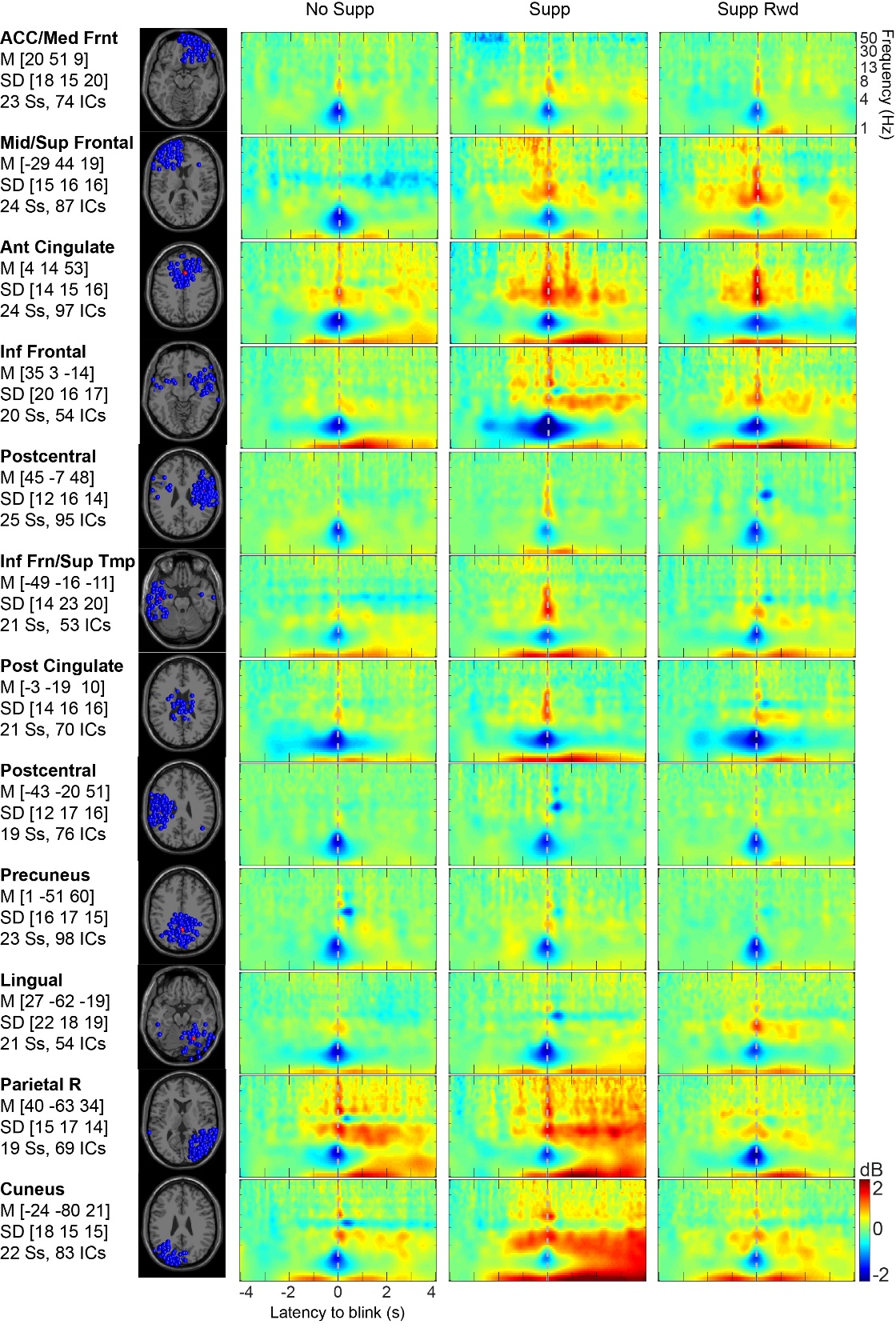


*Figure S9. Event-related spectral perturbation (ERPS) plots showing grand-mean power (across independent components) for each condition (No Supp, Supp, Supp Rwd). The color plotting scheme is in dB, indicating that each pixel power value is converted into ratio by dividing mean power during the baseline period (-4 to -3 s) within the same frequency bin.*


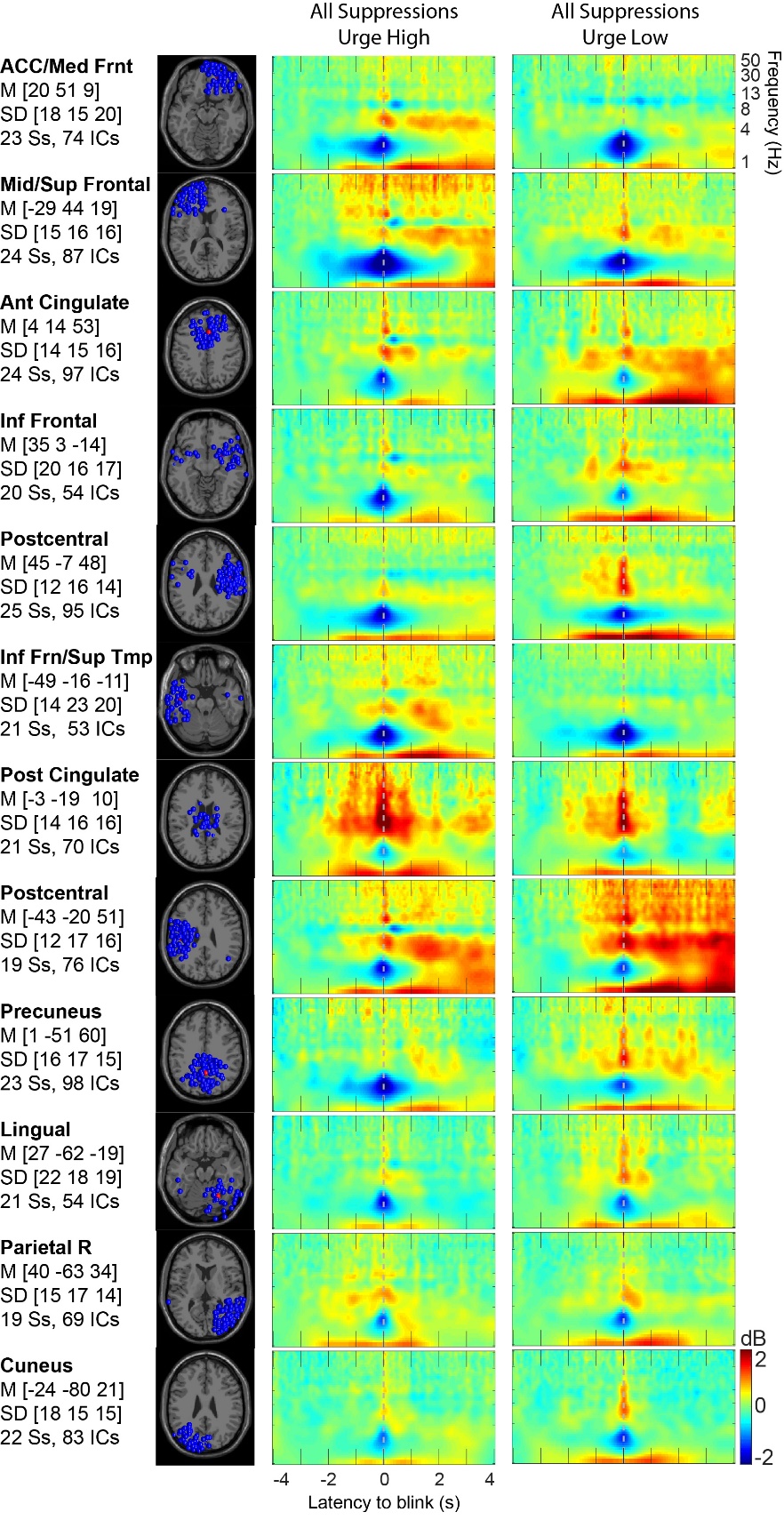


*Figure S10. Event-related spectral perturbation (ERPS) plots showing grand-mean power (across independent components) for each condition (Urge High, Urge Low). The color plotting scheme is in dB, indicating that each pixel power value is converted into ratio by dividing mean power during the baseline period (-4 to -3 s) within the same frequency bin.*


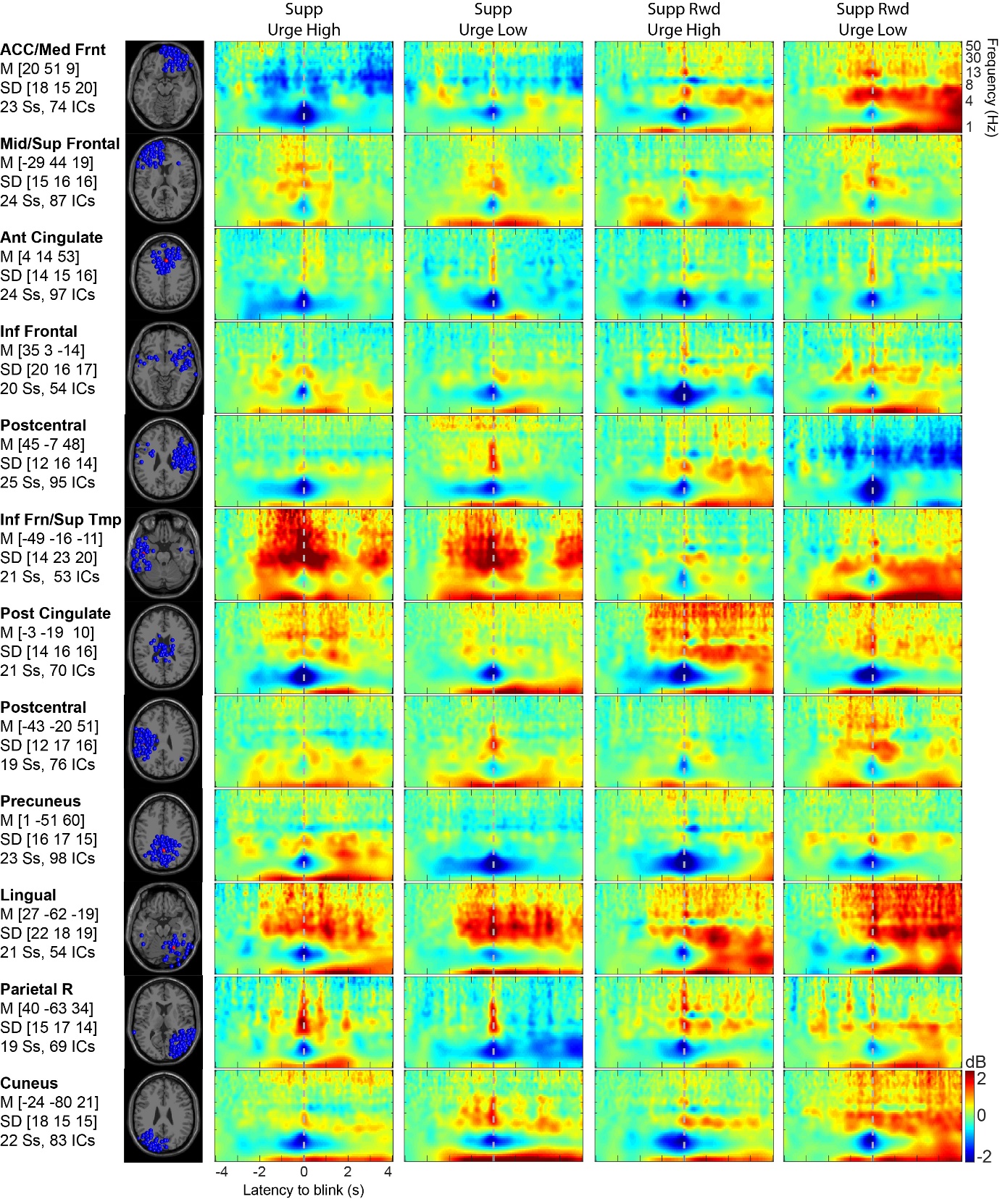


*Figure S11. Event-related spectral perturbation (ERPS) plots showing grand-mean power (across independent components) for each of 2 x 2 combination of conditions (Supp-Supp Rwd vs. Urge High-Urge Low). The color plotting scheme is in dB, indicating that each pixel power value is converted into ratio by dividing mean power during the baseline period (-4 to -3 s) within the same frequency bin.*

# Bibliography

Blum S, Jacobsen NSJ, Bleichner MG, Debener S. 2019. A riemannian modification of artifact subspace reconstruction for EEG artifact handling. Front Hum Neurosci. 13:141.

Calinski T, Harabasz J. 1974. A dendrite method for cluster analysis. Communications in Statistics - Theory and Methods. 3:1–27.

Chang C-Y, Hsu S-H, Pion-Tonachini L, Jung T-P. 2018. Evaluation of artifact subspace reconstruction for automatic EEG artifact removal. Conf Proc IEEE Eng Med Biol Soc. 2018:1242–1245.

Chang C-Y, Hsu S-H, Pion-Tonachini L, Jung T-P. 2019. Evaluation of Artifact Subspace Reconstruction for Automatic Artifact Components Removal in Multi-channel EEG Recordings. IEEE Trans Biomed Eng.

Davies DL, Bouldin DW. 1979. A Cluster Separation Measure. IEEE Trans Pattern Anal Mach Intell. PAMI-1:224–227.

Delorme A, Makeig S. 2004. EEGLAB: an open source toolbox for analysis of single-trial EEG dynamics including independent component analysis. J Neurosci Methods. 134:9–21.

Delorme A, Palmer J, Onton J, Oostenveld R, Makeig S. 2012. Independent EEG sources are dipolar. PLoS One. 7:e30135.

Gabard-Durnam LJ, Mendez Leal AS, Wilkinson CL, Levin AR. 2018. The Harvard Automated Processing Pipeline for Electroencephalography (HAPPE): Standardized Processing Software for Developmental and High-Artifact Data. Front Neurosci. 12:97.

Hsu S-H, Pion-Tonachini L, Palmer J, Miyakoshi M, Makeig S, Jung T-P. 2018. Modeling brain dynamic state changes with adaptive mixture independent component analysis. Neuroimage. 183:47–61.

Kothe CA, Makeig S. 2013. BCILAB: a platform for brain-computer interface development. J Neural Eng. 10:056014.

Kriegeskorte N, Simmons WK, Bellgowan PSF, Baker CI. 2009. Circular analysis in systems neuroscience: the dangers of double dipping. Nat Neurosci. 12:535–540.

Makeig S. 1993. Auditory event-related dynamics of the EEG spectrum and effects of exposure to tones. Electroencephalogr Clin Neurophysiol. 86:283–293.

Mullen TR, Kothe CAE, Chi YM, Ojeda A, Kerth T, Makeig S, Jung T-P, Cauwenberghs G. 2015. Real-Time Neuroimaging and Cognitive Monitoring Using Wearable Dry EEG. IEEE Trans Biomed Eng. 62:2553–2567.

Oostenveld R, Fries P, Maris E, Schoffelen J-M. 2011. FieldTrip: Open source software for advanced analysis of MEG, EEG, and invasive electrophysiological data. Comput Intell Neurosci. 2011:156869.

Palmer J, Kreutz-delgado K, Makeig S. 2016. AMICA: An Adaptive Mixture of Independent Component Analyzers with Shared Components.

Palmer J, Makeig S, Kreutz-Delgado K, Rao B. 2008. Newton Method for the ICA Mixture Model. Proceedings of the 33rd IEEE International Conference on Acoustics and Signal Processing (ICASSP 2008). 1805–1808.

Piazza C, Miyakoshi M, Akalin-Acar Z, Cantiani C, Reni G, Bianchi AM, Makeig S. 2016. An Automated Function for Identifying EEG Independent Components Representing Bilateral Source Activity. In: Kyriacou E, Christofides S, Pattichis CS, editors. XIV Mediterranean Conference on Medical and Biological Engineering and Computing 2016. IFMBE Proceedings. Cham: Springer International Publishing. p. 105–109.

Picton TW, Bentin S, Berg P, Donchin E, Hillyard SA, Johnson R, Miller GA, Ritter W, Ruchkin DS, Rugg MD, Taylor MJ. 2000. Guidelines for using human event-related potentials to study cognition: recording standards and publication criteria. Psychophysiology. 37:127–152.

Pion-Tonachini L, Kreutz-Delgado K, Makeig S. 2019. ICLabel: An automated electroencephalographic independent component classifier, dataset, and website. Neuroimage. 198:181–197.

Plechawska-Wojcik M, Kaczorowska M, Zapala D. 2019. The artifact subspace reconstruction (ASR) for EEG signal correction. A comparative study. In: Świątek J, Borzemski L, Wilimowska Z, editors. Information systems architecture and technology: proceedings of 39th international conference on information systems architecture and technology – ISAT 2018: part II. Advances in intelligent systems and computing. Cham: Springer International Publishing. p. 125–135.

Rousseeuw PJ. 1987. Silhouettes: A graphical aid to the interpretation and validation of cluster analysis. Journal of Computational and Applied Mathematics. 20:53–65.

Winkler I, Debener S, Müller K-R, Tangermann M. 2015. On the influence of high-pass filtering on ICA-based artifact reduction in EEG-ERP. Conf Proc IEEE Eng Med Biol Soc. 2015:4101–4105.
